# Duodenal Lipoma As Upper Gastrointestinal Bleeding Presentation: Case Report and Review of the Literature

**DOI:** 10.7759/cureus.33996

**Published:** 2023-01-20

**Authors:** Mohammed I Mousa, Sara S Al Ghamdi, Ahmad A Alsolmi, Ahmed F Fakhri

**Affiliations:** 1 Internal Medicine/Gastroenterology, King Abdulaziz University Faculty of Medicine, Jeddah, SAU; 2 Internal Medicine, King Abdulaziz Medical City, National Guard Health Affairs, Jeddah, SAU; 3 Medical Intern, King Abdulaziz University Faculty of Medicine, Jeddah, SAU

**Keywords:** piecemeal, endoscopic resection of duodenal lipoma, melena, duodenal lipoma, non-variceal upper gastrointestinal bleeding

## Abstract

Duodenal lipomas (DLs) are rare benign nonepithelial tumors that account for 4% of all gastrointestinal (GI) lipomas. DLs can occur in any part of the duodenum but most commonly arise in the second part of the duodenum. They are usually asymptomatic and discovered incidentally but may present with GI hemorrhage, bowel obstruction, or abdominal pain and discomfort. The diagnostic modalities can be based on radiological studies and endoscopy with the aid of endoscopic ultrasound (EUS). DLs can be managed either endoscopically or surgically. We report a case of symptomatic DL presenting with upper GI hemorrhage along with a review of the literature.

We report a case of a 49-year-old female patient who presented with a one-week history of abdominal pain and melena. Upper endoscopy revealed a single, large pedunculated polyp with an ulcerated tip in the first part of the duodenum. EUS confirmed features suggestive of a lipoma, including an intense homogeneous hyperechoic mass originating from the submucosa. The patient underwent endoscopic resection, with excellent recovery.

The rare occurrence of DLs requires a high index of suspicion and radiological endoscopic assessment to rule out invasion into the deeper layers. Endoscopic management is associated with good outcomes and a decreased risk of surgical complications.

## Introduction

Duodenal lipomas (DLs) are rare benign nonepithelial tumors that account for 4% of all gastrointestinal (GI) lipomas. DLs can occur in any part of the duodenum but most commonly arise in the second part of the duodenum. They are usually asymptomatic and discovered incidentally but may present with GI hemorrhage, bowel obstruction, intussusception, or abdominal pain and discomfort. The diagnostic modalities can be based on radiological studies and endoscopy with the aid of endoscopic ultrasound (EUS). DLs can be managed either endoscopically or surgically. We report a case of symptomatic DL presenting with upper GI hemorrhage along with a review of the literature. 

## Case presentation

**Figure 1:**
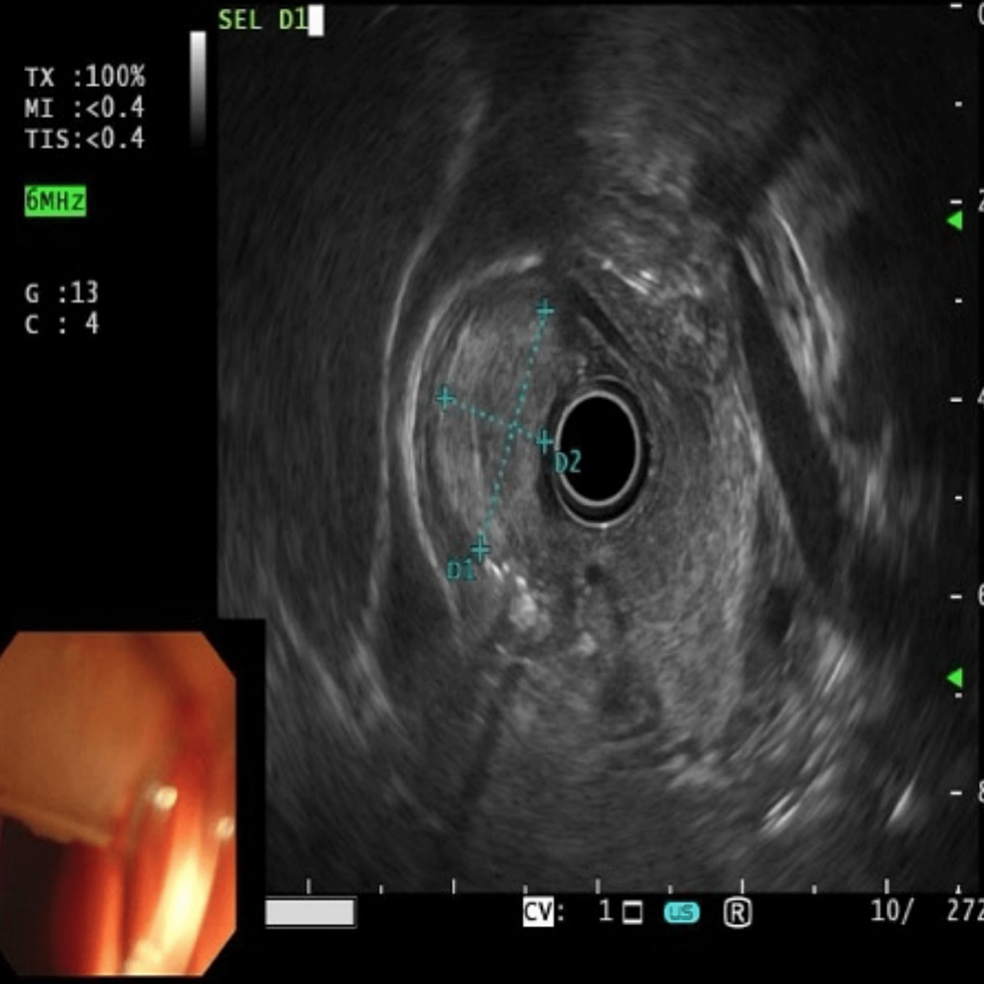
Endoscopic Ultrasound (EUS) showing a mass originated from 2nd mucosal layer

**Figure 2:**
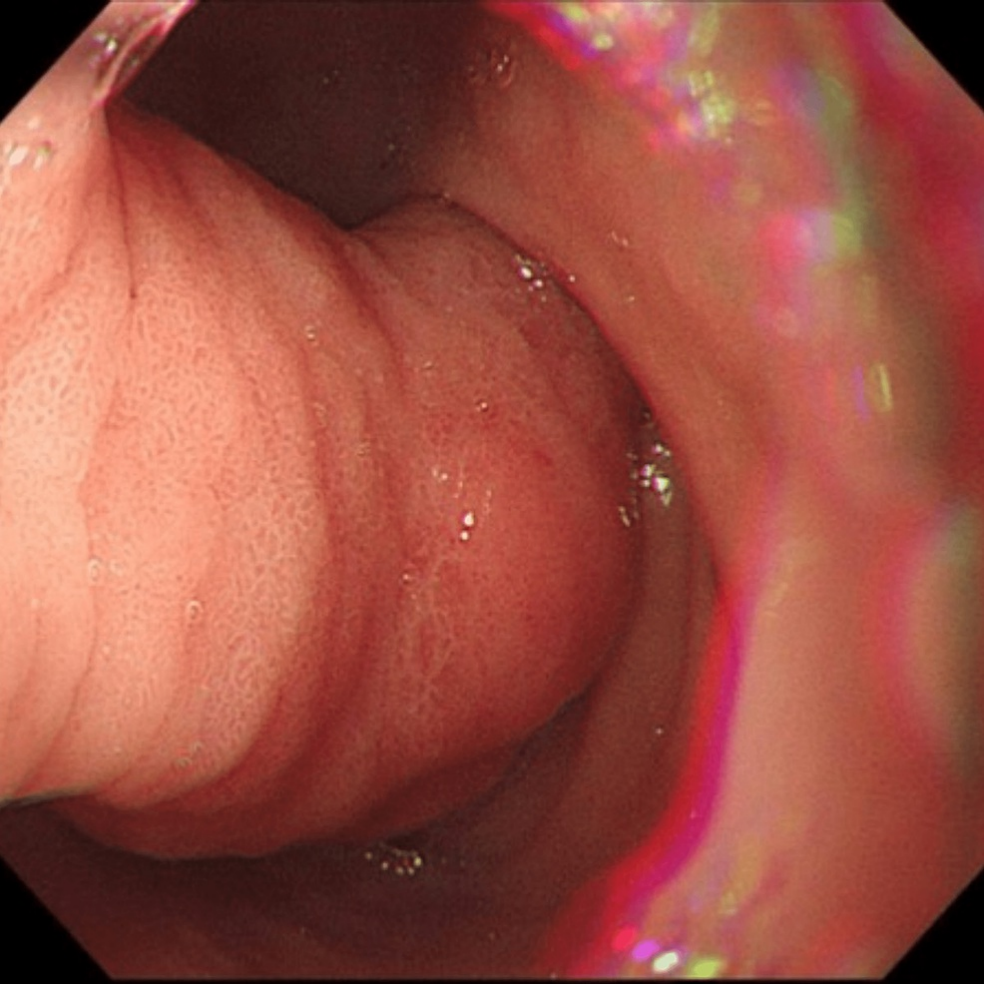
Upper endoscopy revealed a large pedunclated mass at 1st part of the duodenum

**Figure 3:**
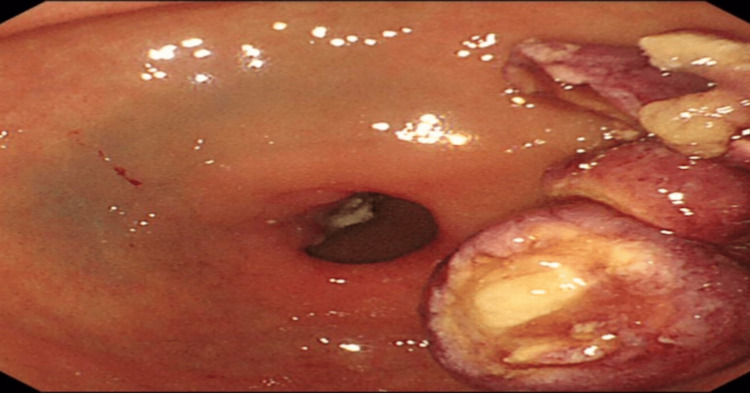
Post piecemeal resection of the polyp

**Figure 4:**
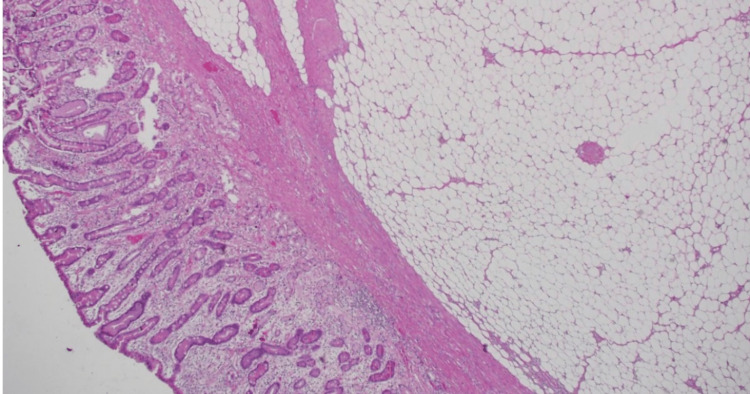
Section shows duodenal mucosa with submucosal circumscribed collection of mature adipocytes consistent with lipoma (Hematoxylin and eosine, 40✖️)

We report a case of a 49-year-old female patient who presented with a one-week history of abdominal pain and melena. EUS confirmed features suggestive of a lipoma, including an intense homogeneous hyperechoic mass originating from the submucosa and it measured 15 mm by 23 mm in diameter (Figure 1). Upper endoscopy revealed an around 2 cm, single large pedunculated polyp with an ulcerated tip in the first part of the duodenum (Figure 2). The patient underwent a colonoscopy followed by a CT abdomen and pelvis to exclude concomitant masses and all were normal. The patient underwent endoscopic resection of the polyp (Figure 3), initially, we injected the base of the polyp with saline and methylene blue to rise it and then we grasped and pulled the polyp into the stomach with a Roth net. Snare (25 mm) mucosal resection was performed; due to its large size, the lesion was debulked from the distal portion and removed in three pieces. The lesion was composed of adipose tissue, consistent with lipoma. A small amount of residual fatty tissue was seen at the resection base; this was left in place. The resected polyp was retrieved with a Roth net. There was no bleeding during the procedure. The patient had an excellent recovery and we planned for the next follow-up upper endoscopy after six months. The histopathological finding is consistent with a lipoma (Figure 4).

 

## Discussion

DLs are rare lesions that may arise in the GI tract, with little literature or consensus on their presentation and management. The most common sites of lipomas in the GI tract are the large intestine (64%), small intestine (26%), duodenum (4%), stomach (3%), and esophagus (2%). There were only 59 published cases of duodenal lipomas, according to a recent systematic review. These were distributed as follows: 20% were in the superior part (D1), 50% were in the descending part (D2), 16% were in the horizontal part (D3), 9% were in the ascending part (D4), and 5% were not defined [1].

The median age at presentation was 62.7 years, with a noticeable female predominance (64.3%). DL can be classified based on its shape appearance as sessile or pedunculated [1]. Most cases (75%) are pedunculated, and the mean size documented throughout the reported cases is approximately 4.1 cm [1, 2, 3].

The pathogenesis of DLs remains unclear; it may develop because of inflammatory stimulation, fat accumulation, and/or abnormal secretion of gonadal hormones in the anterior pituitary [4]. Due to their rarity, these lesions are occasionally underrecognized due to the ambiguity of their presentations, as most small lesions are asymptomatic. In larger lesions (> 2 cm in diameter), 80% of patients become symptomatic. Melena is the most common presenting symptom, accounting for 46.4%, followed by postprandial fullness and abdominal pain. Other presentations reported in the literature include intestinal obstruction [5, 6], intussusception, anemia, and GI hemorrhage [3]. Imaging modalities that could help in diagnosis include computed tomography (CT), which usually shows low-density signals [7, 8]. Magnetic resonance (MR) imaging also provides a detailed characterization of these lesions. On T1-weighted MR images, the lipoma usually appeared as hyperintense. In addition, on T2-weighted images, presented as an intermediate signal intensity; the loss of signal intensity on T1- and T2-weighted fat-suppressed images helped to confirm the diagnosis [6]. EUS can add significant value to the diagnosis of DLs. A case series reported by Chen et al. of eight patients with a pathological diagnosis of DLs stated that all eight lesions appeared as intensive homogeneous hyperechoic lesions [9]. Moreover, EUS can provide valuable details concerning depth and invasion [10].

To our knowledge, this is the second case report of duodenal lipoma in Saudi Arabia, the first of which was treated endoscopically. Daff et al. [8] reported a DL treated with laparoscopic enucleation. Hu et al. reported a retrospective cross-sectional study of 57 patients diagnosed with DLs. Interestingly, they also reported 14 cases of concomitant cancer in other parts of the GI tract. 

Throughout the review, 50% of cases were managed endoscopically, especially in the last 15 years, due to advancements in the endoscopic field. Treatment of GI lipomas is necessary if the lesions cause symptoms, and there is no standard of care to treat symptomatic duodenal lipomas.

Numerous reports have suggested that the endoscopic treatment of GI submucosal tumors is a valid alternative to invasive surgery. Regardless, the treatment choice for GI lipomas remains controversial because it has been documented that the removal of lipomas ≥2 cm in diameter (large lipoma) is associated with a greater risk of hemorrhage and perforation. Multiple studies have shown that endoscopic therapy based on snare removal of small lesions (< 2 cm, including lipomas) is safe and efficacious. In addition, multiple studies have suggested that the assistance of EUS can reduce the risk of perforation [11].

We believe that endoscopic management is a minimally invasive procedure compared with surgical treatment and is considered a safe and effective method for symptomatic large lipomas [12].

The surgical approach is indicated depending on the size of the lesion, location within the duodenum, and risk of perforation with endoscopic treatment [9]. The most commonly documented surgical intervention for duodenal lipomas is laparoscopic transduodenal resection, also known as duodenectomy [9]

## Conclusions

The rare occurrence of DLs requires a high index of suspicion and radiological with endoscopic assessment to rule out invasion into the deeper layers. Endoscopic management in selected cases is associated with good outcomes and a decreased risk of surgical complications.

## References

[1] Pei MW, Hu MR, Chen WB, Qin C (2017). Diagnosis and treatment of duodenal lipoma: a systematic review and a case report. J Clin Diagn Res.

[2] Sou S, Nomura H, Takaki Y, Nagahama T, Matsubara F, Matsui T, Yao T (2006). Hemorrhagic duodenal lipoma managed by endoscopic resection. J Gastroenterol Hepatol.

[3] Yoshii H, Izumi H, Tajiri T, Mukai M, Nomura E, Makuuchi H (2020). Surgical resection for hemorrhagic duodenal lipoma: a case report. Tokai J Exp Clin Med.

[4] Hu ZW, Liang P, Li ZL (2021). Diagnostic value and potential clinical significance of duodenal lipoma based on computed tomography imaging data. Medicine (Baltimore).

[5] Blanchet MC, Arnal E, Paparel P, Grima F, Voiglio EJ, Caillot JL (2003). Obstructive duodenal lipoma successfully treated by endoscopic polypectomy. Gastrointestinal endoscopy.

[6] Kovač JD, Dunjić MK, Bjelović M (2012). Magnetic resonance imaging features of multiple duodenal lipomas: a rare cause of intestinal obstruction. Jpn J Radiol.

[7] Glosser LD, Lombardi CV, Knauss HM, Hopper W, Alalwan A, Stanek S (2021). Treatment of duodenal lipoma with robotic-assisted transverse duodenotomy: a case report of novel approach. Int J Surg Case Rep.

[8] Abu Daff SN, Abu Daff NS (2008). Laparoscopic enucleation of a duodenal lipoma, with review of the literature. Saudi Med J.

[9] Chen HT, Xu GQ, Wang LJ, Chen YP, Li YM (2011). Sonographic features of duodenal lipomas in eight clinicopathologically diagnosed patients. World J Gastroenterol.

[10] Baiss M, Rahali A, Elmajdoubi H (2021). Giant duodenal lipoma: an unusual cause of gastrointestinal bleeding (a case report). Pan Afr Med J.

[11] Yu HG, Ding YM, Tan S, Luo HS, Yu JP (2007). A safe and efficient strategy for endoscopic resection of large, gastrointestinal lipoma. Surg Endosc.

[12] Lee KJ, Kim GH, Park DY (2014). Endoscopic resection of gastrointestinal lipomas: a single-center experience. Surg Endosc.

